# Expert consensus on apical microsurgery

**DOI:** 10.1038/s41368-024-00334-8

**Published:** 2025-01-02

**Authors:** Hanguo Wang, Xin Xu, Zhuan Bian, Jingping Liang, Zhi Chen, Benxiang Hou, Lihong Qiu, Wenxia Chen, Xi Wei, Kaijin Hu, Qintao Wang, Zuhua Wang, Jiyao Li, Dingming Huang, Xiaoyan Wang, Zhengwei Huang, Liuyan Meng, Chen Zhang, Fangfang Xie, Di Yang, Jinhua Yu, Jin Zhao, Yihuai Pan, Shuang Pan, Deqin Yang, Weidong Niu, Qi Zhang, Shuli Deng, Jingzhi Ma, Xiuping Meng, Jian Yang, Jiayuan Wu, Yi Du, Junqi Ling, Lin Yue, Xuedong Zhou, Qing Yu

**Affiliations:** 1 State Key Laboratory of Oral & Maxillofacial Reconstruction and Regeneration, National Clinical Research Center for Oral Diseases, Shaanxi Key Laboratory of Oral Diseases, Department of Operative Dentistry & Endodontics, School of Stomatology, The Fourth Military Medical University, Xi’an, China; 2 State Key Laboratory of Oral Diseases & National Center for Stomatology & National Clinical Research Center for Oral Diseases & Department of Cariology and Endodontics, West China Hospital of Stomatology, Sichuan University, Chengdu, China; 3 State Key Laboratory of Oral & Maxillofacial Reconstruction and Regeneration, Key Laboratory of Oral Biomedicine Ministry of Education, Hubei Key Laboratory of Stomatology, Department of Endodontics, School & Hospital of Stomatology, Wuhan University, Wuhan, China; 4 Department of Endodontics and Operative Dentistry, Shanghai Ninth People’s Hospital, Shanghai Jiao Tong University School of Medicine; College of Stomatology, Shanghai Jiao Tong University; National Center for Stomatology; National Clinical Research Center for Oral Diseases; Shanghai Key Laboratory of Stomatology, Shanghai Research Institute of Stomatology, Shanghai, China; 5 School of Stomatology, Capital Medical University, Beijing, China; 6 Department of Endodontics, School of Stomatology, China Medical University, Shenyang, China; 7 College & Hospital of Stomatology, Guangxi Medical University, Nanning, China; 8 Department of Operative Dentistry and Endodontics, Hospital of Stomatology, Guanghua School of Stomatology, Sun Yat-Sen University & Guangdong Provincial Key Laboratory of Stomatology, Guangzhou, China; 9 Department of Oral and Maxillofacial Surgery, School of Stomatology, Xi’an Medical University, Xi’an, China; 10 State Key Laboratory of Oral & Maxillofacial Reconstruction and Regeneration, National Clinical Research Center for Oral Diseases, Shaanxi International Joint Research Center for Oral Diseases, Department of Periodontology, School of Stomatology, The Fourth Military Medical University, Xi’an, China; 11 Department of Cariology and Endodontology, Peking University School and Hospital of Stomatology & National Center for Stomatology & National Clinical Research Center for Oral Diseases & National Engineering Research Center of Oral Biomaterials and Digital Medical Devices, Beijing, China; 12 Department of Cariology and Endodontology, Peking University School and Hospital of Stomatology, National Center for Stomatology & National Clinical Research Center for Oral Diseases & National Engineering Research Center of Oral Biomaterials and Digital Medical Devices& Beijing Key Laboratory of Digital Stomatology & NHC Key Laboratory of Digital Stomatology & NMPA Key Laboratory for Dental Materials, Beijing, China; 13 Department of Endodontics, Shanghai Ninth People’s Hospital, Shanghai Jiao Tong University School of Medicine; College of Stomatology, Shanghai Jiao Tong University, National Clinical Research Center for Oral Diseases, National Center for Stomatology; Shanghai Key Laboratory of Stomatology, Shanghai, China; 14 Department of Endodontics, School of Stomatology, Capital Medical University, Beijing, China; 15 Department of Endodontics, School of Stomatology, Nanjing Medical University, Nanjing, China; 16 Department of Cariology and Endodontics, The First Affiliated Hospital of Xinjiang Medical University, Urumqi, China; 17 Department of Endodontics, School and Hospital of Stomatology, Wenzhou Medical University, Wenzhou, China; 18 Department of Endodontics, School of Stomatology, First Affiliated Hospital of Harbin Medical University, Harbin, China; 19 Department of Conservative Dentistry and Endodontics, Shanghai Stomatological Hospital & School of Stomatology, Shanghai Key Laboratory of Craniomaxillofacial Development and Diseases, Fudan University, Shanghai, China; 20 Department of Endodontics, School of Stomatology, Dalian Medical University, Dalian, China; 21 Shanghai Engineering Research Center of Tooth Restoration and Regeneration & Tongji Research Institute of Stomatology & Department of Endodontics, Stomatological Hospital and Dental School, Tongji University, Shanghai, China; 22 Stomatology Hospital, School of Stomatology, Zhejiang University School of Medicine, Zhejiang Provincial Clinical Research Center for Oral Diseases, Key Laboratory of Oral Biomedical Research of Zhejiang Province, Cancer Center of Zhejiang University, Engineering Research Center of Oral Biomaterials and Devices of Zhejiang Province, Hangzhou, China; 23 Department of Stomatology, Tongji Hospital, Tongji Medical College, Huazhong University of Science and Technology, Wuhan, China; 24 Department of Endodontics, School and Hospital of Stomatology, Jilin University, Changchun, China; 25 Department of Endodontics, The Affiliated Stomatological Hospital of Nanchang University, Nanchang, China; 26 Department of Endodontics, Affiliated Stomatological Hospital of Zunyi Medical University, Zunyi, China; 27 Jinan Stomatological Hospital, Jinan, China; 28 Hospital of Stomatology, Guanghua School of Stomatology, Sun Yat-sen University, Guangzhou, China; 29 Department of Cariology and Endodontology, Peking University School and Hospital of Stomatology & National Center of Stomatology & National Clinical Research Center for Oral Diseases & National Engineering Laboratory for Digital and Material Technology of Stomatology & Beijing Key Laboratory of Digital Stomatology & Research Center of Engineering and Technology for Computerized Dentistry Ministry of Health & NMPA Key Laboratory for Dental Materials, Beijing, China

**Keywords:** Pulpitis, Root canal treatment, Reconstruction

## Abstract

Apical microsurgery is accurate and minimally invasive, produces few complications, and has a success rate of more than 90%. However, due to the lack of awareness and understanding of apical microsurgery by dental general practitioners and even endodontists, many clinical problems remain to be overcome. The consensus has gathered well-known domestic experts to hold a series of special discussions and reached the consensus. This document specifies the indications, contraindications, preoperative preparations, operational procedures, complication prevention measures, and efficacy evaluation of apical microsurgery and is applicable to dentists who perform apical microsurgery after systematic training.

## Introduction

Root canal therapy is currently the most common and effective method for treating periapical diseases, with a success rate of more than 80%, while the success rate of root canal retreatment can reach 50–80%. Developments in technology, materials, and equipment related to root canal therapy, especially the introduction of dental operative microscopes, have aided in the increase in treatment success rates. However, due to the complexity of the root canal system, the formation of extraradicular bacterial biofilms, and the occurrence of true cysts, some periapical diseases still cannot be cured. In such cases, combined surgical treatment, i.e., endodontic surgery, is needed.^1–8^ Endodontic microsurgery was developed in the 1990s with the application of a dental operative microscope. The magnification and illumination provided by the microscope allow endodontic surgery to be performed using microscopic instruments, ultrasonic tips, and bioactive ceramic materials.^1,9–11^ Three main types of endodontic microsurgery are currently performed: apical microsurgery, periradicular microsurgery, and microscopic intentional replantation. The apical microsurgery is a surgical procedure on the root apex, including osteotomy, root-end resection, root-end preparation, and filling under the microscope. For the cases where apical microsurgery is not feasible, microscopic intentional replantation is indicated, i.e., insertion of a tooth into its alveolus after the tooth has been extracted for the purpose of performing treatment under a microscope, such as root-end filling(s) or perforation repair. Periradicular microsurgery, including root amputation and hemisection, is a surgical procedure for the removal of a root or root of a tooth.^9,12,13^

Compared with traditional apical surgery, apical microsurgery has clear advantages, such as precise identification of root apices, small osteotomy, shallow bell angle of root-end resection, clear exploration of the resected root surface, and accurate root-end preparation. Apical microsurgery is accurate and minimally invasive, produces few complications, and has a success rate of more than 90%.^9,14–55^

However, due to the lack of awareness and understanding of apical microsurgery by dental general practitioners and even endodontists, many clinical problems remain to be overcome, such as the blind expansion of indications, the nonstandardized nature of the operations, the presence of serious complications, and low efficacy.

A search of the literature revealed no relevant studies in Chinese or English, including expert consensuses, guidelines, or specifications, related to apical microsurgery. Neither foreign nor domestic endodontic organizations, such as the American Association of Endodontists (AAE), the European Society of Endodontology (ESE), and the Society of Cariology and Endodontology of Chinese Stomatological Association, have issued expert consensuses, guidelines, or specifications related to apical microsurgery.^56^

To standardize the clinical application of apical microsurgery, the Society of Cariology and Endodontology, Chinese Stomatological Association, has gathered well-known domestic experts, who major in endodontics, periodontics, or oral surgery, to hold a series of special discussions, on the basis of extensive investigations of the research results and clinical experience at home and abroad, we proposed this paper after repeated discussion. The expert consensus aims to guide the orderly, reasonable, and correct clinical implementation of apical microsurgery to improve the level and efficacy of periapical disease treatment and to better preserve natural teeth.^57^

This document specifies the indications, contraindications, preoperative preparations, operational procedures, complication prevention measures, and efficacy evaluation of apical microsurgery and is applicable to dentists who perform apical microsurgery after systematic training.

## Indications

The indications for apical microsurgery include the following: (1) Teeth that still have symptoms and/or positive signs after root canal treatment and retreatment; (2) Inability to gain the coronal access to implement root canal treatment and/or retreatment of the diseased teeth with the presence of symptoms and/or positive signs.^12,13,58–68^

## Contraindications

### Systemic conditions

Patients with systemic diseases or risks should consult corresponding specialists to determine the feasibility of apical microsurgery and the corresponding precautions.^13,61,62,64,69,70^

1. Uncontrolled hypertension, coronary heart disease, and other cardiovascular and cerebrovascular diseases.
2. Elevated risks of secondary infection: infective endocarditis caused by organic heart disease or a state of immunosuppression due to malignant tumors, organ transplantation, or uncontrolled diabetes.
3. Elevated bleeding risk: abnormal coagulation function caused by hemophilia, thrombocytopenic purpura, or other diseases.
4. Existing risk of osteonecrosis of the jaw: previous radiotherapy or injection with intravenous or oral bisphosphonates.
5. Other conditions making the patient unsuitable for surgery, including pregnancy and an inability to cooperate with surgery due to age or mental status.

### Local conditions

If a patient has the following local conditions, the surgeon should carefully evaluate the feasibility of apical microsurgery.^1,12,13,61–64^

. Diseased tooth in the acute inflammatory stage.

. Proximity of the root apex of the diseased tooth to important anatomical structures, such as blood vessels and nerves.

. Difficult lip retraction and obstruction by soft tissues and hard tissues limit the surgical approach.

. Poor oral hygiene and insufficient periodontal support.

. A crown-to-root ratio greater than 1:1 after root end resection or further grinding due to vertical root fracture or external root resorption.

## Preoperative examination

### History and preoperative examination

1. Systemic conditions. The patient’s past medical history, medication history, and allergy history, especially the history of anesthesia-related allergies, should be collected to evaluate systemic health status, to rule out systemic diseases that are not suitable for surgery, and to predict possible complications. Blood pressure should be measured, and a physician should be consulted if necessary.
2. Blood tests. Routine blood test results, clotting time, infectious diseases (hepatitis B, hepatitis C, AIDS, and syphilis), and blood glucose levels should be recorded.
3. Maxillofacial examination. Check whether there is swelling of the maxillofacial region.
4. General oral examination. Examination of temporomandibular joint, width of mouth opening, oral hygiene status, occlusion, oral vestibular depth, muscle attachment, etc. should be performed.
5. Examination of the diseased tooth. The condition of hard tissues, including the shape of the tooth crown, the presence of a restoration, the integrity and marginal adaptation of the restoration, should be assessed.
  The conditions of the periodontal tissues and mucosa, the color and morphological texture of the gingiva and mucosa, the presence of a sinus tract, and the location and source of the sinus tract should be examined. The periodontal probing depth, width of the attached gingiva, condition of the root furcation, and health status of the interdental papilla should be evaluated.
6. Imaging examinations Periapical radiographs and cone beam computed tomography (CBCT) should be obtained. The parallelling projection technique is recommended for periapical radiographs. CBCT can be used to determine the extent of the lesion and to examine the diseased tooth and its anatomical relationship with the surrounding tissues.^71,72^

### Confirming clinical diagnosis and developing treatment plans

A correct diagnosis of the diseased tooth should be made based on the patient’s chief complaints, medical history, and examination results. Systemic and oral health evaluations should be performed, and apical microsurgery should be selected according to the indications.

## Preoperative preparations

### Medical preparations

It is recommended that surgery be performed in a dental clinic with dedicated space and that the clinic room be disinfected. The equipment should include a dental operative microscope and an ultrasonic unit. The instruments should include 45-degree surgical handpiece and long surgical burs; incision, separation, exposure, and suturing instruments; minicurettes; micromirrors; a microexplorer; ultrasonic tips for root-end preparation; and micropluggers. Drugs and other materials include anesthetic drugs, disinfectants, bioactive materials, vasoconstrictors, and stains.

### Patient preparation

Chlorhexidine compound mouthwash should be used, and anti-inflammatory and analgesic drugs should be administered taken orally if necessary. Antibiotics can be used prophylactically when there is a risk of infection.

### Local anesthesia

Anesthesia should cover the diseased tooth plus two neighboring teeth. Infiltration anesthesia is recommended for maxillary teeth, and block and infiltration anesthesia are recommended for mandibular teeth. The local anesthesia is performed according to the standard of the Chinese Stomatological Association “Guideline for oral local anesthesia (T/CHSA 021—2023)”.

### Surgical area preparation

After disinfecting the surgical area, a drape should be applied.

## Surgical procedures

**Fig. 1:**
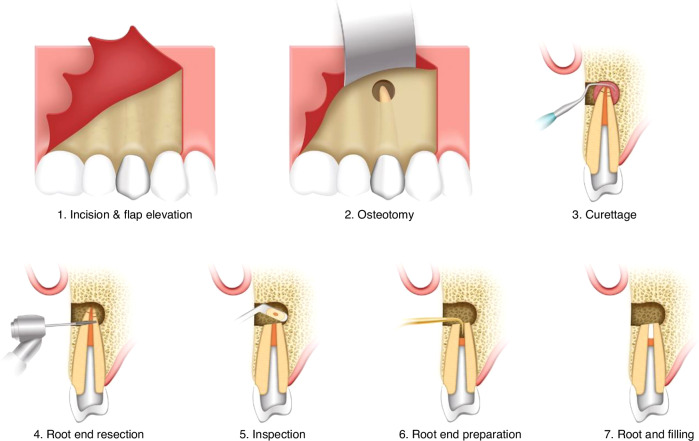
Schematic diagram of clinical operating procedures for apical microsurgery

The clinical operating procedure of apical microsurgery includes seven main steps as shown in Fig. 1.

### Microscope positioning and use

The relative positions of the microscope and the patient should be adjusted so that the operation can be performed under direct microscopic vision. When the resected root surface is inspected and the root end is prepared, the root canal can be observed from a reflected view by micromirror under microscope. Flap incision and suturing should be performed under low magnification, inspection should be performed under high magnification, and other operations should be performed under medium magnification.^10,12,63^

### Flap design

**Fig. 2:**
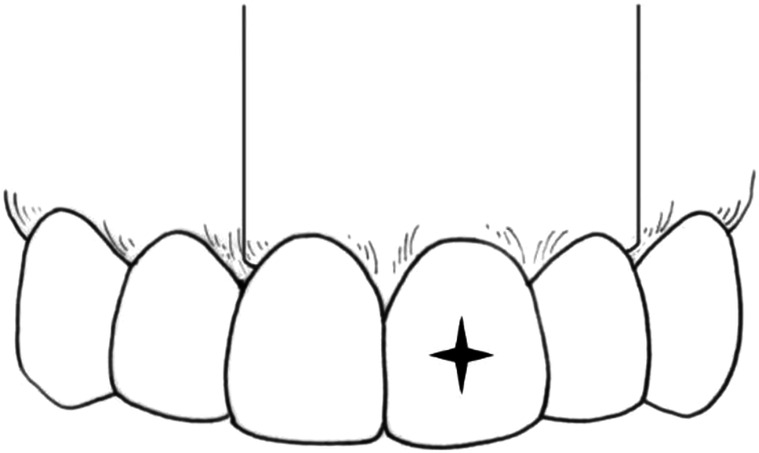
Sulcular rectangular flap with a horizontal incision in the gingival sulcus

**Fig. 3:**
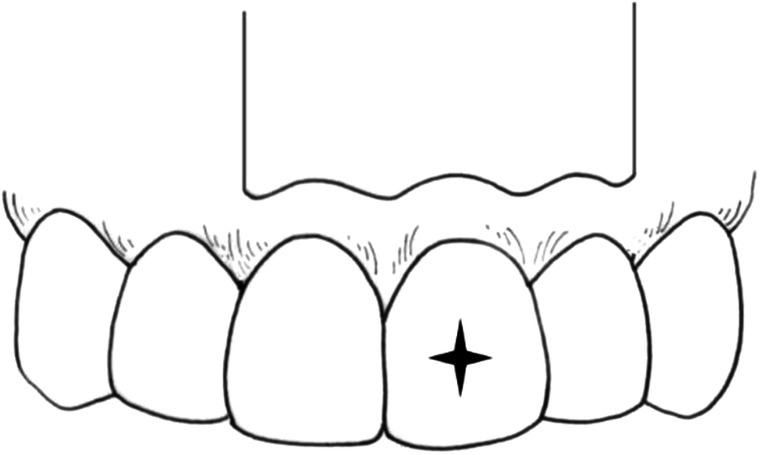
Submarginal rectangular flap with a horizontal incision in the attached gingiva

**Fig. 4:**
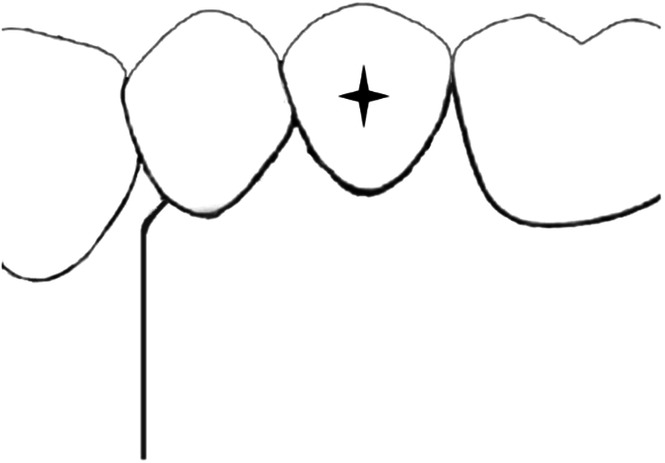
Sulcular triangular flap with a horizontal incision in the gingival sulcus

A full-thickness flap including the diseased tooth and two neighboring teeth should be created with horizontal and vertical incisions; the former should include incisions in the gingival sulcus and attached gingival incisions. A rectangular flap, consisting of a mesial and distal vertical incision and a horizontal incision in the gingival sulcus or the attached gingiva, is usually used for the anterior teeth (Figs. 2, 3); a triangular flap consisting of a mesial vertical incision and a horizontal incision in the gingival sulcus is used for the posterior teeth (Fig. 4). In aesthetically relevant areas, the use of the horizontal submarginal incision or the papilla base incision is recommended, to avoid possible gingival recession from horizontal sulcular incision.^9,73–78^

### Flap incision and elevation

After the surgical blade cuts through the gingiva, mucosa, and periosteum to the bone surface, the full-thickness flap is elevated with a periosteal elevator. Retractors of an appropriate shape should be used to rest on the bone surface, and the flap, lip, and cheeks can be pulled without tension to fully expose the surgical field.

### Root apex positioning

Based on the preoperative CBCT images, the working length of the root canal treatment, the position of a sinus tract, and the alveolar bone eminence at the root, the location of the root apex should be determined precisely.

### Root apex exposure

If the cortical bone at the apical area is destroyed, osteotomy is not necessary. If the cortical bone at the apical area is intact, a 45-degree surgical handpiece with long surgical bur, a trephine, or an ultrasonic osteotome, can be used for osteotomy at the apical area of the diseased tooth to expose the root apex.

### Root end resection, curettage, and inspection

The pathological tissue or foreign bodies in the periapical lesion area should be scraped off.^79–83^

Under sterile water cooling, approximately 3 mm of the root apex is resected, and the cross-section of the root should be positioned perpendicular to the long axis of the root or inclined ≤ 10° in the buccal direction. After resection, the residual pathological tissue is removed, and the resected root surface is smoothed.^84–92^

Epinephrine cotton pellets, ferric sulfate, aluminum chloride, and calcium sulfate can be used for hemostasis via biological effect and/or mechanical compression. The resected root surface should be stained with methylene blue solution, rinsed with normal saline, dried, and inspected under a microscope at high magnification to clarify the presence of vertical root fracture, microleakage, the isthmus, the missing root canal, lateral canals, and perforation, etc.^69,70,93–101^

### Root end preparation and filling

An ultrasonic tip of appropriate diameter and bending direction should be used to lightly “peck” the gutta percha to prepare a Class I cavity coaxial with the root into a minimum of 3 mm depth along the running direction of the root canal while irrigating and cooling. Overcutting of the dentin wall should be avoided, and the microplugger should be used to compact the filling at the bottom of the cavity. The root canal should be cleaned and dried, after which bioactive material is filled into the cavity using the microplugger. The filling material should be compressed layer by layer, and excess material outside of root canal should be removed.^17,101–137^

### Bone crypt treatments

The bone crypt should be rinsed with normal saline to determine whether any foreign bodies have been retained.

### Suturing

The mucoperiosteal flap should be repositioned, aligned accurately, and sutured without tension. Vertical incisions are closed with interrupted sutures, and horizontal incisions are closed with sling or interrupted sutures.

### Suture removal

The time needed for suture removal depends on the condition of the incision. It is generally recommended that sutures be removed 5 to 7 days after surgery.

## Pathological examination

Pathological examination is recommended for removing granulation-like tissue or cystic wall-like tissue after periapical curettage. The pathological examination results should be recorded in the medical records.

## Postoperative management

### Postoperative reactions

After apical microsurgery, some patients may experience mild to moderate pain, swelling, and congestion; severe postoperative reactions are rare.

### Care and medication

After surgery, antibacterial mouthwash should be used to maintain oral hygiene. A cold compress should be applied intermittently for 24 hours in the surgical area, and an intermittent hot compress can be used if swelling still occurs afterward. Analgesics should be taken orally when there is pain. Patients who experience maxillary sinus perforation during surgery should be instructed to sleep with the head facing down, to not blow their nose forcefully, to avoid swimming, and to take antibiotics to prevent infection for 5–7 days after surgery.^138,139^

## Complications

### Surgical area infections

When there are signs of infection, treatment should be administered according to the principles for the treatment of surgical infections.

### Neighboring tooth injury

Injuries of the roots of the neighboring teeth should be avoided during apical microsurgery to the greatest extent possible. In the event of root injury to a neighboring tooth, a sterile cotton pellet should be immediately used to protect the wound surface to avoid contamination. The cotton pellet must be removed before flap repositioning; no special treatment is needed, but the patient should be periodically followed-up.^12,13^

### Maxillary sinus perforation

In the event of maxillary sinus perforation, a cotton pellet tied with a thread can be used to block the perforation to avoid entrance of foreign bodies into the sinus cavity, after which surgery can be continued; if the perforation is large, the use of an absorbable collagen membrane is recommended to repair the maxillary sinus perforation after root-end filling.^140–149^

### Nerve injury

Nerve injury, a serious complication, mostly occurs in the mental nerve, followed by the inferior alveolar nerve. Accurate preoperative positioning and effective intraoperative protection of the neurovascular bundle are required to avoid irreversible damage.^150^

### Other

Other complications, including vascular injury, soft tissue laceration, incision dehiscence, and surgical site infection, should be treated according to standard surgical principles.

## Efficacy evaluation

### Follow-up

Clinical and imaging examinations are regularly performed 3, 6, 12, and 24 months after surgery. For patients who still have periapical lesions 1 year after surgery, follow-up should be conducted annually; observation should continue until 4 years after surgery.

### Efficacy evaluation

Surgical efficacy should be preliminarily evaluated 1 year after surgery, and finally determined 4 years after surgery.^16–18,25–30,50^ Apical radiographs should be routinely taken. For patients who still have symptoms and for whom preoperative CBCT was taken, the scan can be used to evaluate the healing status of the periapical lesions. Successful efficacy is indicated if the diseased tooth has no pain or swelling, there is good healing of soft tissues, there are no sinus openings, and there is no loss of function, and if imaging examinations show that the periapical lesions have disappeared or shrunk. Surgical failure is considered if the diseased teeth have clinical symptoms and signs and the imaging examination shows no change or expansion of the periapical lesions. For teeth without clinical symptoms and signs but whose imaging results reveal indeterminate healing, continued observation of the teeth is recommended.^15,151–159^

## Medical records

The medical records, including clinical examination, radiologic images, consultant, informed consent, prescription, surgical procedure, pathological examination results, and follow-ups, should be standardized and saved.

## Conclusions

Following the biological concepts, i.e. complete debridement, tight sealing of root canal system, and conservation of dental tissue, the apical microsurgery, combined with the magnification and illumination provided by the dental operate microscope with the proper use of micro instruments, ultrasonic retrotips and bioceramics as root-end filling materials, can treat the endodontic origin diseases precisely and less traumatically with high success rate. More and more natural and healthy teeth have been preserved successfully. There are many technical changes that added to the evolution of apical microsurgery, including piezoelectric surgery, static navigation, dynamic navigation, augmented reality-guided surgery, and robot-assisted surgery.^67,98,141,160–198^
